# Assessing the Impact of COVID-19 Vaccination Programs on the Reduction of COVID-19 Cases: A Systematic Literature Review

**DOI:** 10.5334/aogh.4484

**Published:** 2024-07-22

**Authors:** Brightwell Sibanda, Budi Haryanto

**Affiliations:** 1 Department of Environmental Health, Faculty of Public Health, Universitas Indonesia, Depok, West Java, Indonesia; 2 Research Center for Climate Change, Universitas Indonesia, Depok, West Java, Indonesia

**Keywords:** COVID-19, vaccine efficacy, reduction cases, severity illness, systematic review

## Abstract

*Background:* Vaccination is the most effective way to prevent serious illness and death from COVID-19 among the various preventive interventions available.

*Objective:* This review aimed to assess the actual effectiveness of COVID-19 vaccines in curbing the transmission and incidence of COVID-19 cases, to examine the role of different vaccine types in controlling the COVID-19 pandemic, as well as to identify the key factors influencing the efficacy of COVID-19 vaccines in containing the spread of the virus.

*Methods:* The suggestions made by the PRISMA Framework were adhered to. To find the publications for the 2020–2023 timeframe, searches were performed through the PubMed databases, EMBASE, Scopus, and ProQuest. For the review, 17 reports satisfied the inclusion requirements. Ad26.CoV2.S or ChAdOx1-S, Gam-COVID-Vac(GAM), Sinovac Life Sciences Co., Oxford–AstraZeneca, Pfizer–BioNTech, and viral vector vaccines are among the vaccines that act on various variations. They dealt with the Delta, B.1.1.519, Omicron, and Alpha variations.

*Findings:* Vaccinations against various Variants resulted in fewer COVID-19 infections, fewer deaths, and fewer hospitalizations. The emergency of the Delta variant, persons over 60, and vaccine hesitancy were the main issues affecting the effectiveness of COVID-19 vaccinations in containing the virus’s spread.

*Conclusion:* The collective evidence strongly supports the conclusion that COVID-19 vaccination plays a crucial role in mitigating the spread of the virus and reducing the severity of illness among those who contract the virus.

## Introduction

Vaccination is the most effective way to prevent serious illness and death from COVID-19 among the various preventive interventions available [1]. It further significantly reduces the risk of severe illness, hospitalization, and death associated with COVID-19, particularly among high-risk populations and vulnerable individuals. It has the best chance of providing a secure and efficient means of fostering immunity and halting the transmission of disease in order to combat this pandemic [2]. High vaccination rates within a population can lead to herd immunity, providing indirect protection to those who cannot be vaccinated, including individuals with certain medical conditions or weakened immune systems. More so, life-saving discoveries like the development of effective vaccinations have contributed to the global elimination and prevention of numerous infectious diseases [3].

Following the COVID-19 epidemic, the World Health Organization (WHO) and several scientific teams are developing vaccinations [4]. Several nations have quickly attained significant coverage of COVID-19 vaccinations after the vaccines were originally approved in late 2020 [5]. Vaccination is essential in enabling the reopening of businesses, schools, and other public institutions, hence fostering economic recovery and stability, as it stops the virus from spreading. In the field of public health, vaccinations are a remarkable accomplishment, and the creation of COVID-19 vaccines has been named the most significant scientific breakthrough of 2020 [6]. Vaccination increases the resilience of healthcare systems by supporting public health initiatives, which makes it possible to effectively manage COVID-19 cases and other healthcare requirements. During the first phase of the vaccination campaign, four vaccines—two mRNA-based vaccines produced by Pfizer-BioNTech and Moderna, and two vaccines based on an adenovirus vector produced by Oxford-AstraZeneca and Janssen—were approved for use against coronavirus disease 2019 (COVID-19) in the European Union [7].

The number of thromboembolic events in vaccinated people is no higher than the number seen in the general population, the European Medicines Agency (EMA) declared on March 11, 2021 [8]. Vaccination provides a layer of protection for high-risk groups, including the elderly, individuals with pre-existing medical conditions, and frontline healthcare workers, reducing their vulnerability to severe COVID-19 outcomes.

COVID-19 is a disease caused by the Severe Acute Respiratory Syndrome Coronavirus 2 (SARS-CoV-2) [9]. Patients with chronic comorbidities like heart disease, immunological defects, diabetes, and airway disease have been the majority of those affected by the illness since it was declared a pandemic by the WHO in March 2020. It is a deadly, severe, and extremely contagious illness that spreads mostly by direct touch and respiratory secretions between people [10]. The global health catastrophe caused by the COVID-19 pandemic has resulted in massive losses of human life as well as hitherto unheard-of socioeconomic difficulties. On December 1, 2019, Wuhan, China, reported the first incidence of COVID-19, which later spread throughout the world. The WHO declared on March 11, 2020, that COVID-19 qualifies as a pandemic [11]. As of September 2023, there were over 6 million deaths and over 770 million confirmed cases which had been reported worldwide [12].

Despite the widespread administration of COVID-19 vaccines, uncertainties persist regarding their overall effectiveness in reducing the transmission and spread of the virus. Variations in vaccine efficacy, the emergence of new viral variants, and differences in vaccination coverage globally have contributed to the complexity of assessing the true impact of vaccination programs in containing the pandemic. Consequently, there is a critical need to comprehensively evaluate and synthesize the existing research to determine the actual efficacy of COVID-19 vaccines in curtailing the spread of the virus.

## Methods

The PRISMA Framework recommendations was performed in this systematic review [13]. It covered the identification part, screening, and eligibility, and included development of a search strategy to identify relevant literature published in English. The search strategy included the use of PubMed databases, EMBASE, Scopus, and ProQuest to identify the articles for the period 2020 to 2023. Keywords and controlled vocabulary terms related to the research topic were used to conduct the search. The mentioned terms were searched from the title, abstracts, and keywords of the publications. The primary outcome was the reduction of COVID-19 cases because of COVID-19 vaccination programs. For upholding high quality of the documents, all duplicates were thoroughly checked. Duplicates were detected using Rayyan and later exported to Zotero software. Abstracts of the articles were further scrutinized to ensure quality and relevance of the included articles in the review process.

## Results

**Figure 1:**
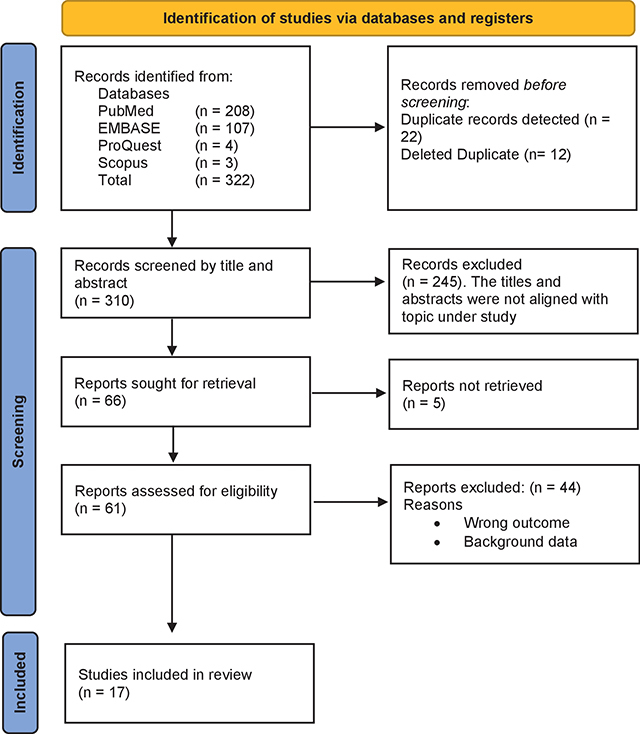
Study profile.

A database search turned up 322 papers, as shown in Figure 1 of the study profile. Out of the recognized articles, 12 were removed after 22 duplicate records were found, leaving 310. Next, it was sorted by abstract and title, which resulted in the removal of 245 items. Out of the 66 reports that were requested to be retrieved, 5 were not retrieved, leaving 61 reports. Following that, a comprehensive text screening was done, which resulted in the removal of 44 more reports, leaving only 17 for the review.

A selection of the features of the publications that were part of this systematic literature evaluation are displayed in Table 1. From the 17 articles that were presented, the following are the journals: BMC Medicine [14], International Journal of Infectious Disease [15], Lancet [16], BMC Infectious Disease [17], Infection Control and Hospital Epidemiology [18], JAMA Network [19], International Journal of Environmental Research and Public Health [20], Scientific Reports [21–23], Clinical Microbiology and Infection [24], Epidemics [25], Morbidity and Mortality Weekly Report [26], Infectious Disease Modelling [27], Open Forum Infectious Diseases [28], and Vaccines [29, 30].

**Table 1:** General characteristics of the included articles.

AUTHOR AND YEAR	JOURNAL	DATABASE	STUDY DESIGN	STUDY TIME FRAME	COUNTRY	POPULATION CHARACTERISTICS	N
Liu Y et al. 2023 [14]	BMC Medicine	PubMed	Epidem. and Economic Model	2020–2021	England	Age structure and non-pharma interventions	27 countries
Bello-Chavolla et al. 2023 [15]	Intl J Infectious Diseases	PubMed	Retro. Cohort Design	2020–2021	Mexico	18 years and older	793,487
McNamara LA et al. 2022 [16]	Lancet	PubMed	Ecological Analysis	2020–2021	USA	18 years or older	49 states
Pattni K et al. 2022 [17]	BMC Infectious Diseases	PubMed	SIR model	2021	England	Demographic data	2,691,413
Dunbar E et al. 2021 [18]	Infection Control and Hospital Epidem.	PubMed	Clinical trial	2020	United States	Health workers	Not provided
Steele MK et al. 2022 [19]	JAMA Network	PubMed	Modelling study	2020–2021	United States	Age groups of 18–49, 50–64 and older	69 million infections
Shim E, 2021 [20]	Intl J Env Research and Pub Health	PubMed	Age-structured model	2021	South Korea	Age groups	70% coverage
Homan T et al. 2022 [21]	Scientific Reports	PubMed	Retro Cohort study	2021	Italy	16 years and older	4.09 million
Kayano T et al. 2021 [22]	Scientific Reports	PubMed	Transition model	2021	Japan	All vaccinated individuals	Not specified
Tonnara G et al. 2022 [23]	Clinical Microbio and Infection	PubMed	Retro Observ study	2021	Republic of San Marino	12 years and older	32,126
Chen X et al. 2022 [24]	Scientific Reports	PubMed	SIR model	2020–2021	United States	Not specified	Not specified
Childs L et al. 2022 [25]	Epidemics	PubMed	Model of COVID-19 Infection	2020–2021	Canada	Age groups	Not specified
Link-Gelles R et al. 2023 [26]	MMWR	PubMed	Model	2022–2023	United States	Age groups 18 years and older	29,175
Mancuso et al. 2021 [27]	Infectious Disease Modelling	Embase	Two strain-group Mechanistic model	2020–2021	United States	Groups of vaccinated and unvaccinated	Total pop in the community
Perez-Then et al. 2023 [28]	Open Forum Infectious Diseases	Embase	Case – Control study	2021	Dominican Republic	18 years and older	1,078
Spreco et al. 2022 [29]	Vaccines	Embase	Case – Control study	2021– 2022	Sweden	18 years and older	1,760,000
Wang et al. 2022 [30]	Vaccines	Embase	Structural Nested Mean Model	2021	United States	Vaccinated population	4.55 million

In this study, the study designs include Epidemiological and Economic Model [14], Retrospective Cohort Design [15, 21], Ecological Analysis [16], SIR model [17, 23], Clinical trial [31], Modelling study [19], Age-structured model [20], Transition model [22], Model of COVID-19 Infection [25], Model [32], Two-strain, two-group Mechanistic model [27], Case–Control study [28, 29], and Structural Nested Mean Model [30]. This Systematic Literature Review included the following countries: England [21, 22], the United States [16, 19, 23, 27, 30–32], Mexico [15], South Korea [20], Italy [21], Japan [22], the Republic of San Marino [24], Canada [25], the Dominican Republic [28], and Sweden [29]. Specifically, the population features identified in this systematic literature review are summed up as follows: The age categories covered by the following articles were 18 years old and older [15, 19, 28, 29, 32].

We predict that a lower vaccination effectiveness will be associated with higher healthcare costs and fewer total Quality-Adjusted Life Years (QALYs) saved as compared to the absence of vaccination. Increased Incremental Cost-effectiveness Ratios (ICERs) relation to Gross Domestic Product (GDP) per capita [14] and a decline in effectiveness in individuals over 60 with a predominance of Delta [15] are the anticipated outcomes of this. Younger people often showed lower VE (vaccine effectiveness), but VE was consistently >70% for those over 60 who had received all vaccinations, including the Gam-COVID-Vac vaccine [24]. The efficacy of treatment against SARS-CoV-2 infection declined in individuals 60 years of age or older when the Delta variant predominated [15]. After three to four months of immunization, the efficacy against death from the Delta variant decreased to 48.47% (CI95% 34.82–53.97) after seven to eight months [21].

The distribution of a particular vaccine among different age groups would therefore have an impact on its efficacy [17]. Moreover, changing the susceptibility of vaccinated individuals was how immune evasion—like that seen with Omicron—reduced the efficacy of vaccination [14]. A certain proportion of the population may not receive the vaccination due to vaccine reluctance. Studies reveal that vaccination hesitancy is a common phenomenon in the US, where some people show resistance to getting vaccinated due to a lack of trust in government agencies, a perceived risk-versus-benefit analysis, or a particular religious belief [23].

The effectiveness of vaccinations in preventing newly discovered coronavirus mutations is a matter of further concern. As an example, vaccinations are less effective against the Delta version, and it is yet unknown how well they can protect against infection with the Omicron variant [23]. Therefore, given the anticipated obstacles to obtaining vaccines (such as production or shipment delays), it seems prudent to administer the first dose now and postpone the second dose until the necessary shipments are obtained to achieve a vaccination program with full coverage, which calls for two doses and a 28-day interval between doses [25].

## Discussion

The deployment of several types of COVID-19 vaccines marks a significant milestone in public health, showcasing unprecedented scientific collaboration and innovation. These vaccines include mRNA vaccines (Pfizer-BioNTech, Moderna), viral vector vaccines (AstraZeneca, Johnson & Johnson, Sputnik V), protein subunit vaccines (Novavax), inactivated virus vaccines (Sinopharm, Sinovac), DNA vaccines (ZyCoV-D), and virus-like particle (VLP) vaccines (Medicago). mRNA vaccines represent a groundbreaking technology, utilizing genetic instructions to prompt an immune response. Viral vector vaccines use a modified virus to deliver genetic material from the coronavirus, stimulating immunity. Protein subunit vaccines present harmless pieces of the virus to the immune system, while inactivated vaccines use a killed version of the virus. DNA vaccines, a novel approach, deliver DNA sequences encoding antigens, and VLP vaccines mimic the virus structure without causing disease. This diverse array of vaccines was developed rapidly due to global cooperation, extensive funding, and previous research on related viruses. Their variety ensures a broader immunological approach, catering to different population needs and logistical challenges. The simultaneous use of multiple vaccine types exemplifies adaptability in combating the pandemic and paves the way for future vaccine development and distribution strategies.

Three important findings have come from this study. First, full vaccination with COVID-19 vaccines has been shown to be efficacious in fighting a broad range of variants. Second, the primary result of vaccination efficacy was the prevention of COVID-19 instances, which in turn decreased hospitalization and, eventually, COVID-19-related fatalities. The third important finding is determining the critical elements affecting vaccination efficacy in keeping COVID-19 contained. According to this study, receiving all COVID-19 vaccines has shown to be efficacious in preventing the spread of several variants, including the Omicron, Delta, B.1.1.519, and Alpha. This was consistent with a study conducted in the United States [23] which hinted that getting a full immunization in two doses appears to have a greater effect. Similarly, additional research [33–37] did not identify the kinds of variations that were acted upon.

Results from two other trials [18, 19] show that for both, the VE of complete vaccination (95% Cl) was 95%. The results align with those of a different study [36], which reported that the vaccinations’ efficacy increased after the second dosage was given, reaching 95% (95% CI, 91–97%) for mRNA-1273, 87% (95% CI, 69–95%) for ChAdOx1, and 92% (95% CI, 90–94%) for BNT162b2. The vaccinations differed slightly from the previously utilized Pfizer/BioNTech vaccines, mRNA-1273 and BNT 162b2. In both investigations, mRNA-1273 and BNT 162b2 were prevalent. A 95% success rate in preventing COVID-19 was demonstrated in a different trial by [37] BNT 162b2, with a 95% credible interval spanning from 90.3 to 97.6.

Along with a VE of full vaccination (95% Cl), the same study [17] also discovered that Pfizer-BioNTech had 83.9% and Oxford-AstraZeneca had 64%. These results were consistent with another trial [17] conducted in England, which showed that Pfizer-BioNTech was 83.9% (95% credible range [82.1, 85.6]) effective against infection while Oxford-AstraZeneca was 64.0% (95% credible interval [61.4, 66.5]) effective for two doses. This demonstrated a certain degree of COVID-19 vaccination efficacy [38].

An additional study revealed that the vaccination program implemented in Malaysia had successfully reduced the risk of illness. The distribution of COVID-19 cases according to immunization status in August 2021 was evident from the fact that most cases that were reported involved those who had not received the vaccine [39]. A study undertaken in the United States supported the results, indicating that two-dose schedules of the Moderna and Pfizer-BioNTech mRNA vaccines showed a noteworthy level of efficacy in avoiding hospitalizations caused by COVID-19 in a hands-on evaluation carried out across 21 hospitals [40]. Furthermore, studies on vaccine efficacy have shown that when exposed to the Delta variation of the virus rather than the Alpha variant, the effectiveness of a single dose of either BNT 162b2 or ChAdOx1 is decreased [41]. However, after receiving two doses of the vaccine, the effectiveness significantly rises and gets closer to the levels seen against the Alpha variant [42].

Additionally, Tonnara et al. confirms that, in the sub-analysis limited to the Alpha-VoC prevalent period, adjusted VE was 96.2% overall and 97.3% in those receiving Gam-COVID-Vac [24]. This shows that the vaccination has a high level of efficacy against the Alpha version in terms of preventing and lowering COVID-19 instances. Another study conducted in Japan revealed that the peak daily frequency of new cases would have decreased by 73% if the vaccination program had started 14 days earlier than it did [43]. The total number of infections that would have occurred under the early schedule scenario would have been 26,149 (with a 95% confidence interval of 24,354–27,952), compared to 98,368 (4 times) more than the number of infections that were actually recorded.

Moreover, Table 2 provides overwhelming evidence that the vaccine’s primary effect was to prevent COVID-19 cases, which in turn reduced hospitalization and, ultimately, COVID-19-related deaths. The vaccine also demonstrated the highest level of efficacy in preventing SARS-CoV-2 infection and COVID-19-related hospitalization. This analysis is consistent with a study conducted in Saudi Arabia wherein the group of people who did not receive vaccinations had worse outcomes in terms of longer hospital stays, more admissions to the intensive care unit, and a need for mechanical ventilation (*p* < 0.001) [44].

**Table 2:** Effectiveness of COVID-19 vaccines in curbing the transmission and incidence of COVID-19 cases.

AUTHOR	TYPE OF VACCINE	VARIANT	VACCINATION STATUS ASSESSMENT METHOD	VE OF FULL VACCINATION (95% CL)	OUTCOME
Liu Y et al. 2023 [14]	mRNA vaccines Viral vector vaccines	Omicron	Not specified	mRNA vaccines – 0.85/0.7 Viral vector vaccines –0.75/0.65	High disease burden minimized. Deaths reduced.
Bello-Chavolla et al. 2023 [15]	BNT162b2 mRNA vaccine	Delta B.1.1.519	Self-reported by the evaluated person	80.34	Efficacy in combating SARS-CoV-2 infection. Preventing hospitalization.
McNamara LA et al. 2022 [16]	Not specified	Not specified	Data from the CDC	Not specified Oxford–AstraZeneca = 64%	Reduction in COVID-19 cases, reduced emergency department visits and hospital admissions.
Pattni K et al. 2021 [17]	Oxford–AstraZeneca Pfizer-BioNTech	Delta	Combined Intelligence for Population Health Action data	Pfizer-BioNTech = 84%	Reduces susceptibility to COVID-19 infection.
Dunbar E et al. 2021 [18]	Pfizer/BioNTech	Not specified	Not indicated	95%	Decrease in COVID-19 infections.
Steele MK et al. 2022 [19]	mRNA-1273 BNT 162b2	Delta	As reported to the CDC Sept 2021	95%	Averts hospital admissions, illnesses, and fatalities in the US.
Shim E, 2021 [20]	AstraZeneca-Oxford and Pfizer mRNA	Delta	Not specified	55–56%	Re = 1.3, it would prevent 47% of symptomatic infections and lower the attack rate 9.2% to 4.9%.
Homan T et al. 2022 [21]	Ad26.CoV2.S or BNT162b2 or mRNA-1273 or ChAdOx1-S	Alpha Delta	Surveillance data of Apulia Region	Overall effectiveness at 92.6%	Prevented illnesses. High efficiency against hospitalization for those who were fully vaccinated.
Kayano T et al. 2021 [22]	Gam-COVID-Vac(GAM)	Alpha	Data from RSM Health System	Gam-COVID-Vac(GAM) = 97.3%	Effective at preventing SARS-CoV-2 infection.
Tonnara G et al. 2022 [23]	Gam-COVID-Vac or BNT162b2	Alpha	RSM Health System	Gam-COVID-Vac = 84 % or BNT162b2 = 16%	Prevention against infection and protection against hospitalization.
Chen X et al. 2022 [24]	Sinovac Life Sciences Co, Vero cell, Pfizer or BioNTec, AstraZeneca	Not specified	Self-reported participant’s vaccination history	31%	Reduction of symptomatic illness. Decreased the likelihood of COVID-19-related hospitalization.
Childs L et al. 2022 [25]	They were kept at constant during the study	Not specified	Vaccination coverage data	They were kept at constant during the study.	Reduction in infections.
Link-Gelles R et al. 2023 [26]	mRNA	Omicron BA.5–and XBB/XBB.1.5	Self-reported	Provided per age group, no overall VE was provided	Provided additional protection against symptomatic XBB/XBB.1.5.
Mancuso et al. 2021 [27]	Pfizer or Mordena	Alpha (B.1.1.7) Wild type strain Delta	Bloomberg COVID-19 Vaccine Tracker Open Data	67% against the Delta variant. Not specified for other variants	Reduction of COVID-19-related cases and deaths.
Perez-Then et al. 2023 [28]	Inactivated Vaccine (Corona Vac)	Ancestral Delta	Data collected through the questionnaire.	31%	Provided a moderate level of protection against symptomatic SARS-CoV-2 infections, prevented COVID-19-related hospitalization.
Spreco et al. 2022 [29]	BNT162b2 mRNA	Alpha Delta	Data from the country wide health information systems	Odds ratio in the vaccinated group was 2.2	Did not offer much protection against COVID-19 cases.
Wang et al. 2022 [30]	BNT162b2 or mRNA	Original strain	Data from JHU Resource Center and CDC	90%	Notable decrease in disease prevalence within the US.

It’s suggested that if the immunization program had a higher transmission potential, Re = 1.3, 47% of symptomatic infections would be prevented and the attack rate lowered from 9.2% to 4.9%; this study also showed the vaccines’ ability to effectively prevent symptomatic infections [18]. This demonstrates the effectiveness of the immunizations in preventing COVID-19. Findings of Shim (2021) and Wells (2024) showed a decline in COVID-19 cases [20, 45]. This observation was consistent with the results reported in Tonnara et al. (2022) that Gam-COVID-Vac [84%] and BNT 162b2 [16%] were generally successful at preventing SARS-CoV-2 infection [24]. Furthermore, Shim (2021) noted that the age range of 40–59 years had the biggest decline in terms of relative terms (55–56%) [20].

As was already mentioned, a number of parameters were shown to be strongly dependent on vaccine efficacy. When persons older than 60 years old had a prevalence of the Delta variant, the efficacy of mRNA-1273 against that variant declined; it was agreed that older adults (over 60) also affected the effectiveness of the vaccine, since their age reduced the vaccines’ ability to prevent coronavirus sickness [15]. Another study conducted in the Republic of San Marino supported the findings of previous authors who suggested that VE declined in young adults but steadily remained over 70% for those over 60 who had received all recommended vaccinations [46]. Both the general population and individuals who received the Gam-COVIDVac vaccination showed this tendency [47].

This study found that treating SARS-CoV-2 infection was less successful in those with diabetes, those 60 years of age or older, and in situations where the Delta variant predominated [15]. This is in line with another study done in Mexico, which discovered that individuals 60 years of age or older or those presenting with diabetes had a lower efficacy in terms of susceptibility to SARS-CoV-2 infection and COVID-19-associated mortality among the cohort of fully immunized subjects [48]. Additionally, this was in line with a study of Sadarangani et al. that examined senior citizens [49]. Furthermore, the study in Mexico (2023) observes that a reduction in vaccine efficacy in a single dosage was a result of the Delta fluctuation [15], Delta’s contribution to lowering vaccination efficacy during its peak of popularity [19, 21]. These results on the reduction of vaccine efficacy due to the Delta variant were also consistent with the study in Mexico (2023), which showed that protection against the Delta variant was lowered when the Delta variant was more common [48]. It’s agreed that the Delta variation in Scotland has caused a decline in effectiveness [50]. The results of this review were supported by a different study, which showed that the estimated VE against symptomatic Delta infection gradually decreased over time after two doses of the COVID-19 vaccine (with at least one mRNA vaccine) [51]. The VE of full vaccination (95% Cl) for Sinovac Life Sciences Co., Vero Cell, Pfizer or BioNTec, and AstraZeneca was found to be 31% in this study as well. However, this is much lower than the anticipated 95%.

## Conclusion

This comprehensive analysis of the scientific literature on the effects of COVID-19 vaccination campaigns has shed important light on how well vaccinations work globally to lower the incidence of COVID-19 cases, especially when it comes to battling various variants. The conclusion that COVID-19 immunization is essential for limiting the virus’s transmission and lessening the severity of disease in those who get it is highly supported by the body of available data. Moreover, vaccinations have shown to be highly effective at preventing infection, and among those who have had vaccinations, breakthrough infections are frequently linked to fewer hospital admissions, fewer deaths, and milder symptoms. Furthermore, the effectiveness of the vaccine was strongly influenced by several factors, including older age and the prevalence of Delta variations. This emphasizes how important vaccination campaigns are to enhancing global vaccine equity, monitoring vaccine effectiveness and variants, effecting public health measures, ensuring good communication, and investing in research and development.
